# Unpacking the interaction between foreign language learners’ emotion, cognition, and activity in the flipped classroom in higher education: A *perezhivanie* perspective

**DOI:** 10.3389/fpsyg.2022.1005237

**Published:** 2022-10-11

**Authors:** Lili Qin, Lan Yao, Yinxing Jin

**Affiliations:** ^1^Department of World Languages, Dalian University of Foreign Languages, Dalian, China; ^2^Ganzhou Middle School, Ganzhou, China; ^3^Foreign Language College, Hainan Normal University, Haikou, China

**Keywords:** *perezhivanie*, emotion-cognition-activity, dramas, sociocultural theory, flipped classroom

## Abstract

Few studies have investigated learners’ emotional experiences and the interactions between emotion, cognition, and activity in the flipped foreign language classroom (i.e., a mixed teaching mode that combines in-class teaching and off-class self-directed learning). This study, from the perspective of *perezhivanie* (a concept from sociocultural theory), addressed these research gaps by exercising a longitudinal narrative study on a total of 32 Chinese-as-the-first-language university students of English who attended a 15-week English course with this teaching design. Among them, eight focal students were randomly selected for further evidence of the characteristics of the interactions between emotion, cognition, and activity. The results showed that the participants experienced more of positive emotions than negative emotions in the flipped classroom (FC) context, which supports the efficacy of the pedagogy. But most importantly, complex interactions between emotion, cognition, and activity were revealed. Generally, (negative) positive emotion, cognition, and activity were interconnected; however, what is also evident is that learners’ emotions either promoted or inhibited their cognitive functions, and positive and negative emotions did not necessarily correspond to positive and negative activities, respectively. This is due to the presence of dynamic, developmental, and historical sociocultural mediators in learners *perezhivanija*, be it teacher, peers, technology, teaching materials, teaching activities in an FC, or the learners’ previous English learning anecdotes, etc.

## Introduction

All the learning processes unify cognition and emotion including second-language learning. However, researchers in the field of second-language acquisition (SLA) have primarily focused on learners’ cognitive performance, leaving their emotional experiences less explored (Swain, 2013; Dewaele and Li, 2018). Among the limited number of research addressing learners’ emotions in the field, a large majority of them targeted only a single emotion, such as anxiety, enjoyment, burnout, and boredom (Dewaele and MacIntyre, 2014; Jin and Zhang, 2019, 2021; Derakhshan et al., 2021; Li, 2022), despite the facts that second-language learning is a rather complex process whereby many positive and negative emotions can be experienced even in the same time window and that it is the positive or negative emotionality that motivates or demotivates learners over a long learning trajectory. Methodologically, research on emotions has heavily relied on using Likert-type scales. Albeit warranting a large dataset (Jiang and Dewaele, 2019; Li, 2021, 2022), the downsides of this research paradigm are the dichotomy of emotions into either positive or negative (Wang et al., 2021), and only a single form of emotional manifestation is attended to. Therefore, qualitative data are needed with its advantages of including the characteristics of typicality, complexity, and integrity, although the sample size is small.

In addition, though FC teaching as an alternative to replace teacher-led instruction in varying educational settings (van Alten et al., 2019), particularly in the second-language (L2) domain (Mehring and Leis, 2018), has advantages such as more frequent inter-student interactions before and after class (Roehl et al., 2013), flexibility (Buechler et al., 2014), and increased student engagement (Chetcuti et al., 2014; Malik et al., 2018), it also imposes new challenges on teachers and students alike. For example, the use of information technology in FC teaching can make learners feel difficult to concentrate (Qin et al., 2022). They also face greater pressure to engage in more interactive activities in the in-person part of the FC teaching (Malik et al., 2018). All new challenges are likely to influence foreign language learners’ emotions; consequently, it is necessary to conduct a more in-depth discussion on learners’ emotional experiences and influencing factors of learners’ emotion in FC teaching of foreign languages.

By far, scarce studies have explored how learners’ emotions form and develop in the FC from a *perezhivanie* (unity of emotion, cognition, and social activities from SCT) perspective to understand how learners interpret and emotionally relate to the teaching environment, which may shed some light on relevant studies. Thus, this study aims to explore the developmental process of learners’ emotions in the context of the FC through the lens of *perezhivanie*. To achieve this goal, we adopted a multi-dimensional narrative approach (Patton, 1990) to triangulate the findings by deploying written narratives at two time points (at the beginning and end of the semester), as well as on a longitudinal weekly basis lasting for 15 weeks. The synchronic and diachronic narrative data provided evidence not only on learners’ emotional states and its dynamic changes but also on the dialectical interactions between learners’ emotion, cognition, and activities in the FC context.

## Literature review

This section provides interpretations of related concepts such as sociocultural theory, *perezhivanie* and drama, as well as a brief overview of existing research on learner emotion.

### Sociocultural theory

Sociocultural theory (SCT) rooted in cultural-historical psychology established by Vygotsky and his colleagues (Lantolf and Thorne, 2006). Although SCT literally includes “social” and “cultural,” it is a theory of neither society nor culture, but one of mind that explains the laws of human beings’ mental functioning developed from social relationships and cultural artifacts (Qin et al., 2019; Qin, 2021, 2022; Qin and Ren, 2021). The philosophical origins of SCT are from the 18th and 19th centuries by Germany philosophers Kant’s and Hegel’s dialectics, as well as Marx and Engels’ works on sociology and economics critically drawing on Feuerbach’s tenets of materialism (Lantolf and Thorne, 2006).

Traditional psychology theories are keen to separate cognition from emotion (Vygotsky, 1987). However, SCT advocates their integrated and interactive contributions to the development of mental functioning by proposing the concept of *perezhivanie*. Originating from the Russian word *perezhivat*, *perezhivanie* refers to “how an individual is aware of, interprets, and affectively relates to a certain event” (Vygotsky, 1994, p. 341). It continuously develops in a dynamic manner since a very young age as individuals gain lived experiences in the real world. *Perezhivanie* thus implies the effect of the immediate and past sociocultural environment on individuals, more specifically social relationships and social activities (Vygotsky, 1994). Having said that, it must be noted that not all social relationships and social activities influence individuals’ development according to SCT but only those “dramatic” ones (also known as dramas) that cause one’s internal emotional conflicts and create critical *perezhivaniya* (Lantolf and Swain, 2019). Research from an SCT perspective typically uses critical *perezhivaniya* (plural form, or *perezhivanija*) as units of analysis, which automatically attends to tripartite interactions among emotion, cognition, and social environment (Roth and Jornet, 2013) and combines the past, the present, and the future (Veresov, 2017).

### Perezhivanie

*Perezhivanie* refers to an individual’s lived or emotional experience, which is subjective thinking and feeling of the environment, that is, “how the individual is aware of, interprets, and affectively relates to a certain event” (Vygotsky, 1994, p. 341). As a unit of analysis, *perezhivanie* represents the dialectical unity of emotion and cognition. An individual’s *perezhivanie* exerts effects on the individual’s social activity and eventually builds the trajectory of development (Vygotsky, 1987; Golombek and Doran, 2014). Therefore, *perezhivanie* covers both objective environmental characteristics and subjective personal characteristics. The difference between *experience* and *perezhivanie* is that the former is a complete discrete event that can be divided according to time and be recalled through memories, while the latter is the ongoing conversion of social activities from the social to the individual. It is a dynamic and continuously updated unit that involves the interaction between situations, cognition, and emotions relative to the individual (Roth and Jornet, 2013). Vygotsky (1998) believes that the concept of *perezhivanie* can be used to analyze the influence of the social and cultural environment on the process of individual development. In short, individuals experience the current environment through their past *perezhivanija* and then form their new *perezhivanija*, and the current *perezhivanija* will have an impact on how individuals experience the new environment in future.

### Drama

Vygotsky (1997) believed that researchers need to trace the individual history, that is, their past experience. Veresov (2017, p. 59) pointed out that social relations in the individual’s past experience would bring individual development, and not every social relation can be developed into individual psychological functions but only those “dramatic” social relations can. That is to say, only those social relations that influence the individual’s emotions can be used as the source of development (Lantolf and Swain, 2019). In a word, dramas are those events that cause the individual’s internal emotional conflicts, which is in line with the genetic law. The relationship between drama, *perezhivanie*, and development is that drama which brings rich emotions forms an individual’s critical *perezhivanie*, thus influencing the individual’s trajectory of development (Fleer et al., 2017). From a *perezhivanie* perspective, despite the same environment, some would feel it dramatic, thus gaining the opportunity to develop, while others may not feel the environment special to personal development.

### Learners’ emotion in sociocultural theory

Previous learners’ emotion research from psychological perspectives would focus on specific types of emotion and attempt to explore the relation between emotional variables and other quantifiable variables (Khajavy et al., 2018; Li et al., 2020; Pawlak et al., 2020). However, emotion research from the SCT perspective does not delve into the constituent variables of emotion but records and describes emotional experience, traces the process of emotional development, and explores the interaction between learners and the sociocultural environment from a holistic view. For example, Swain et al. (2015) used narrative interviews to build a multilingual learning history of a participant, Grace and constructed a developmental trajectory by understanding her lived emotional experience (*perezhivanie*). Sampson (2020) followed 47 Japanese first-year college students and asked them to write down their feelings during English class every week. After quantifying the qualitative data, it was found that the sources of positive and negative emotions are mainly classroom activities, classmates, identity, teacher, and lesson. This kind of diachronic tracking of emotions and feelings of a group can record learners’ emotional states and sources of fluctuations and have important reference value for emotion research in SLA.

### Learners’ emotion studies in flipped classroom

An FC is commonly referred to as the process of flipping what is traditionally carried out in the classroom to an independent homework activity before class. As such, lessons involve problem-solving and higher order thinking tasks, which are traditionally assigned to subsequent homework activities (Mehring, 2016, 2018; Låg and Saele, 2019). Vitta and Al-Hoorie (2020) defined FC as “…*involves presentation of new content to learners to be independently studied before class, and then class time is devoted to reinforcing and engaging with the ‘flipped’ content*.” In other words, in the FC, the in-person time aims to help students solve problems and to engage students in collaborative and hands-on activities (Bergmann and Sams, 2014; Mehring, 2018). The online time, an extension of in-class learning, makes use of digital resources to support students’ learning linguistic knowledge and intercultural knowledge (Hung, 2015, 2017; Chen Hsieh et al., 2017). In the traditional classroom, teachers inculcate knowledge in students, who often learn in a passive way. Instead, within the pedagogical framework of the FC, teachers assist in and guide learners through their learning process and thus allow greater learner autonomy (Evseeva and Solozhenko, 2015; Adnan, 2017).

The FC indeed can provoke both positive and negative learners’ emotions; however, so far, little research in EFL teaching has focused on both dimensions of emotions. Only Kruk and Pawlak (2022) discussed this systematically in second-language virtual classrooms, but mainly from a dualistic perspective, dividing positive and negative emotions into two non-interfering dimensions and only described characteristics of each, respectively. In the existing research on both sides of learners’ emotions in the FC, most of it has been teaching and learning in science classrooms. For example, Jeong et al. (2016) conducted a study in a general science course with sophomores in a university in Spain and concluded that students’ perception toward an FC included both negative (boredom and fear) and positive (fun and enthusiasm) emotions. González-Gómez et al. (2017) performed a comparative study between the traditional classroom (TC) and FC in a general science classroom with undergraduate students and concluded that in contrast to the TC, the FC aroused more of positive emotions than negative ones. However, in EFL classrooms, there are more studies focusing on one dimension of learners’ emotion in the FC, be it positive or negative. For example, Pan et al. (2022) inspected the effect of Massive Open Online Course (MOOC) and FC on EFL learners’ foreign language-speaking anxiety and attitude toward English learning and concluded that participants in both groups had positive attitudes toward technological-based instructional environments. Gok et al. (2021), however, did not find the FC triggered significantly higher anxiety, suggesting the complexity of the antecedents of emotions, and many factors might moderate the effect of pedagogical approaches on learners’ emotions.

Another strand of research focused on the effect of participating in classroom activities on emotions in the FC context. For example, Gok et al. (2021) found that pre-class preparation and in-class group work could alleviate learners’ anxiety. Abdullah et al. (2020) found that participating in the well-designed in-class and out-of-class activities provided students many opportunities to improve their self-confidence and significantly reduced their anxiety due to a creative, safe, comfortable, and encouraging learning environment forged by the teaching approach. Malik et al. (2018) evaluated the effectiveness of the FC approach by examining the relationship between the FC and student engagement at physical, behavioral, and emotional levels. The findings indicate that the FC improves learners’ physical and cognitive engagement; however, no improvement in their emotional engagement was observed.

Above all, we note that from an SCT perspective, the interaction between emotion, cognition, and activity should be examined if deeper understanding of learners’ emotion in the FC is to be achieved. More importantly, the cognitive, affective, and social interactions of individuals should be integrated as a whole. Although studies on neurosciences have proved the link and inseparability between cognition, emotion, and human behaviors due to mental trauma (Sarr-Jansman and Rowberry, 2018; Malaei et al., 2022; Quadt et al., 2022), and researchers from education (Yob, 1997) and psychology (Greenberg and Safran, 1987; Eich and Schooler, 2000; Schnitzspahn and Phillips, 2016; Belkhir, 2020) also noted the gap, only a few studies found this trend in SLA. However, most previous studies discussing the three components in one study in SLA were on second-language teacher education. For example, Tasker et al. (2010), Golombek and Doran (2014), Johnson and Worden (2014), Golombek (2015), Johnson and Golombek (2016), Johnson (2018, 2021), Agnoletto et al. (2021), discussed novice language teachers’ development due to emotional and cognitive dissonance during the practices of learning to teach. Even fewer studies focused on the interaction between the second-language learner’s emotion, cognition, and activities in a holistic way (unity of individuals and society). Only Moeller (2021) discussed about the significance of integrating feeling and thinking to optimize language learning, claiming that if positive emotions are activated, learners would put more efforts to language learning and show greater sense of efficacy. However, by far, no empirical studies on the interaction of the three components of *perezhivanie* have been found implemented among second-language learners in the FC.

In line with the previous discussion, the concept of *perezhivanie* views individuals’ emotions, cognitions, and activities (social activities engaged in) as inseparable and also as a unity of the dialectical relationship between the three (Swain, 2013; Lantolf and Swain, 2019). Furthermore, we note that existing research rarely describes the dynamic changes over time in learners’ emotions in the FC. Therefore, we argue that in this context, there is a need for a longitudinal study on learners’ emotions from a *perezhivanie* perspective. Based on this argument, the present study is guided by the following questions:

(1) What are learners’ emotional experiences in the context of the FC?
(2) In what ways are learners’ emotion, cognition, and activity combined in the context of the FC?
(3) What are the dramas (unit of analysis for *perezhivanie*) that cause the interaction between emotion, cognition, and activity?

## Methodology

### Context

The study was carried out in an EFL lesson using an FC approach at a Northeastern China university. The teacher for this lesson was a novice teacher with no prior experience in foreign language teaching in higher education. The classes for this lesson lasted 17 weeks. Review and final examination were conducted in weeks 16 and 17, and we only collected data from week 1 to week 15. In each week, students had one face-to-face session (90 mins), either *integrated* or *listening and speaking class* (two modules of EFL), supplemented by self-directed online learning through a platform called Unipus (90 mins). In addition, the students could communicate with the teacher through WeChat, a real-time communication tool like Facebook Messenger and email, if they had any questions.

### Participants

The total participants were 32 sophomore undergraduates in semester 4, majoring in a foreign language other than English, but all took English as a foreign language course at a Northeast China university. There were 28 female and four male students, with an average age of 19.6 (*SD* = *1.1*) years, who consented to participate in a written narrative task in weeks 1 and 15, respectively (weeks 16 and 17 were not included in the research because they were the time for review and final examination). Among them, eight students were randomly extracted from low-, medium-, and high-English level subgroups of the total sample achieved by allotting the 32 students to the three groups in terms of their scores in a national-level English examination. These focal students, representing different foreign language backgrounds and different levels of English proficiency, additionally accomplished narrative journals on a weekly basis over 15 weeks of the semester. We present the narrative diary data of the eight focal students after their consent. Table 1 reports in details the demographic information of eight focal students who consented the narratives to be included in the study anonymously.

**TABLE 1:** Participants’ demographic information.

Pseudo name	Gender	Major language	English learning duration	CET 4^1^ grade	English proficiency subgroup
Rita	Female	Russian	12 years	548	High
Jenny	Female	Japanese	11 years	528	High
Linda	Female	Japanese	11 years	517	Medium
Ryan	Female	Spanish	14 years	502	Medium
Zoey	Female	Arabic	13 years	449	Low
Vicky	Female	Russian	11 years	485	Low
Jim	Male	Japanese	11 years	489	Low
Jack	Male	Spanish	15 years	414	Low

^1^A national-level English test in China called College English Test. Its equivalence to other international tests is illustrated at: https://ucsantabarbaraextension.zendesk.com/hc/en-us/articles/360001614547-English-Language-Requirements-for-International-Programs. 550 + is nearly equivalent to IELTS 6.5. 425 is a threshold of passing grade which is roughly equivalent to IETLS 4.5. An introduction to College English Test in China can be found at https://wenr.wes.org/2018/08/an-introduction-to-chinas-college-english-test-cet.

### Instruments and procedures

Narrative is a way of using language and other signs (images, gestures, etc.) to produce a coherent account that posits an interconnection between the past, the present, and the future events (Dick et al., 2017). Emotion and cognition are both developed from social activities (Gorbatkov, 2002; Golombek and Doran, 2014); therefore, cognition, emotion, and activity are inevitably unified in their narratives. Narrative analysis is interested in broader interpretive frameworks used by both the participant and the researcher to make sense of particular incidents in the individual’s lives, which is compatible to the “drama,” unit of analysis for *perezhivanie*, meaning the “dramatic social relations or events” that caused individual development (Fleer et al., 2017, p. 59). Researchers use narrative analysis to understand how participants construct stories from their own personal experiences, which contain the interaction of emotion, cognition, and activity, suiting for the current study. Johnson and Golombek (2002, p. 4) proposed that “the conceptualization of narrative inquiry in Dewey’s (1916; 1920; 1933) educational philosophy, which, at its core, argues that we are all knowers who reflect on experience, confront the unknown, make sense of it, and take actions.

A written narrative was adopted in this study. The total of 32 participants performed written narrative tasks in their native language, Chinese (minimally 200 words), in weeks 1 and 15, respectively. In week 1, they reported their language learning experiences prior to the current semester, including pre-college experiences. In week 15, they reported their emotional experiences in in-class learning, including the emotions experienced at the time points of presenting, answering questions, discussing, participating in other activities, and independent learning (watching online courses, etc.). In other words, the purposes of the narrative study on the participants were to understand their English learning experiences prior to, at the beginning of, and during FC learning, respectively (question 1). In total, 17,783 Chinese characters were collected.

To investigate the interaction between emotion, cognition, and activity (question 2) and locate the dramas that caused the change of *perezhivanija* or interaction of the three elements (question 3) in the FC context, weekly written narrative reports were collected from eight focal participants who consented to submit their reports. The contents pertained to pre-class online learning experiences, in-class activities, and after-class activities (see Table 2). In total, 29,319 Chinese characters were collected from this weekly narrative task.

**TABLE 2:** Aspects to be covered in eight focal students’ written narratives.

Situations	Narrative aspects
**Before class:** video watching, self-directed learning on UNIPUS, learning the resources shared by the teacher or peers or found by the participants themselves, etc.	Emotions and cognition in ongoing events
**In class:** interactions with the teacher and peers, classroom activities, etc.	
**After class:** review, homework, peer interactions, and other activities etc.	

### Data analysis

Data analysis was conducted on NVivo 12 plus. The purposes were to elicit the participants’ emotional experiences in the FC context and the co-concurrences of emotion, cognition, and activities (or actions taken). For the former, we not only coded the emotions explicitly expressed by the participants in their self-narratives but also those hidden in the lines. For the latter, we adopted *perezhivanie* as the unit of analysis, that is, we identified the episodes in the participants’ self-narratives that contain dramas causing the interaction of emotion (E), cognition (C), and activity (A) and tried to make sense of the ways in which they co-existed. The first and the second authors independently analyzed the data. They achieved a high inter-rater consensus (90%). The two researchers discussed to solve the disagreements that occurred between them.

As shown in Table 3, we coded narrative data based on the three compositional elements of *perezhivanie*—emotion, cognition, and activity. As *perezhivanie* is historical and developmental as discussed before, we also coded learners’ language learning dramas or dramatic events that caused learners’ *perezhivanija* to change, either in the history (before college), prior to the term of the current study, or during the process of the term (in college). To get a clearer picture of FC teaching, we also traced those dramas that happened both in face-to-face classes, where the teacher presented in-person, and outside of the classroom where learners self-guided their learning with the help of assigned online digital resources, where the teacher presented through online support. In addition, we intended to clarify that the coding of emotion, cognition, and activities, respectively, are specifically justified, as given in the following text.

**TABLE 3:** Coding scheme of the analysis of leaners’ written narratives.

Time and space	Dramatic events/Dramas and examples	*Perezhivanie* (definitions for the three elements as seen in the paragraph below)
**Before College**	English learning histories or historical ***dramas*** that caused change of learner *perezhivanie*, i.e., learners’ interactions with teachers, classmates, friends, parents, technology etc.	**Emotion** Such as positive emotions including enjoyment, gratitude, interest, and excitement; negative emotions fear, anxiety, embarrassment, sense of difficulty, and boredom etc. **Cognition** As defined in Vygotsky (1978), Belkhir (2020), cognition of English learning in this study means how a leaner believes an activity or task (as social interactions) as a mediator to English learning. **Activity** English learning activities that learners participated in.
**In College**	**Out-of-class:** English learning stories or ***dramas*** that caused change of learner *perezhivanie*, i.e., learners’ interaction with video watching, autonomous learning, the content of video, time management, the use of technology, etc. **In-class:** English learning stories or ***dramas*** that caused change of learner *perezhivanie*, i.e., learners’ interaction with teachers, classmates, friends, parents etc., including sociocultural mediators such as group discussion, group presentation, error correction, assessment, the use of technology, etc.	

### Coding of learners’ emotions toward flipped classroom

As discussed earlier regarding learners’ emotion from the SCT perspective, the researchers do not specify positive or negative emotions. Therefore, for learners’ emotion in this study, we coded all kinds that emerged out of the data, including positive emotions and negative emotions (for details, see Table 4).

**TABLE 4:** Coding scheme of varieties of learners’ emotions emerged in narrative data.

Level 2 code	Level 1 code	Definition and example
Positive emotions	enjoyment	when a learner recorded feeling a positive, activating emotion arising from ongoing learning activities or tasks (Dewaele, 2021; Li et al., 2021), i.e., “I enjoyed watching videos at face-to-face class.”
	easiness	When a learner recorded feeling comfortable or relaxed, or free from worry or pain in learning something or participating in a task (Cambridge Dictionary Online), i.e., “The teacher made extensive use of multimedia in the class, creating a relaxed learning atmosphere.”
	interest	Foreign language learning interest is a state of wanting to learn or know something out of curiosity. Interest is feeling and commitment to something or activity without command (Shanty, 2019), i.e., “I think the videos we watched in the class were quite interesting.”
	Sense of achievement	English learning achievement is defined as the perceived and assessed part of a learner’s mastery of abilities and subject materials as estimated with legitimate and valid tests (Joe et al., 2014), i.e., “The process of preparing for the presentation and presenting it in class made me feel great.”
	enthusiasm	When a learner recorded a feeling of energetic interest in English or activity and a desire to be involved in learning English (Yusriyah et al., 2021), i.e., “I’m always passionate about learning English.”
	Sense of expectation	When a learner recorded the feeling that good things are going to happen in the future in English learning (Leal et al., 2017), i.e., “I was looking forward to taking English classes every week.”
	gratitude	when the learner feels obliged to do something out of gratefulness to the teacher’s timely support (Wilang, 2022), i.e., “I am very grateful to the teacher for her encouragement before the CET 6 exam.”
	excitement	When a learner recorded feeling excited to do something in learning English (Cambridge Dictionary Online), i.e., “The teacher said ‘very good’, which made me excited and happy for the whole class.”
	surprise	When a learner recorded feeling surprised to do something in learning English (Cambridge Dictionary Online), i.e., “I was surprised to hear the different ideas I was exposed to.”
	Self Confidence	Learner’s self-confidence in using the L2, operationally defined in terms of low anxious affect and high self-perceptions of L2 competence (Clément et al., 1994; Listyani and Tananuraksakul, 2019), i.e., “After I finished the presentation, the teacher encouraged me and affirmed my English pronunciation and Mandarin, which made me feel more confident about myself.”
	Sense of fulfillment	When a learner recorded feeling happiness because of doing what he/she intended to do in language learning (Cambridge Dictionary Online), i.e., “The learning methods and the English videos shared by the teacher in Wechat group are very useful. I have a full sense of gain.”
	Less reluctance	When a learner recorded feeling more willingness than reluctance to participate in English learning activity (grounded theory), i.e., “Compared to last semester, at least in this semester I was less reluctant when I completed my study tasks.”
	Feeling of novelty	When a learner recorded feeling happiness with learning something new about English (Mather and Plunkett, 2012), i.e., “I felt that the teacher was particularly enthusiastic, and the teaching contents she used were more novel and fresher compared to my previous English teachers.”
Negative emotions	Nervousness^1^	when the learner recorded feeling nervous of answering questions in class (Weitoft and Rosén, 2005; Hishikawa et al., 2019), i.e., “I was nervous when the teacher asked questions.”
	Boredom	When a learner recorded feeling bored as a negative, deactivating emotion arising from ongoing learning activities or tasks, and anxiety as a negative, activating emotion evoked by envisioned results related to future learning outcome or performance (Li et al., 2021), i.e., “I think it’s a bit boring to learn contents on *UNIPUS*.”
	Anxiety	when a learner stated feeling a negative, activating emotion evoked by envisioned results related to future learning outcome or performance (Jin et al., 2021; Li et al., 2021), i.e., “Sometimes I also felt anxious, like when I was about to take an exam, but I haven’t started to review or I did not know anything yet.”
	Sense of difficulty	when a learner recorded feeling difficult of doing some tasks or learning something, i.e., “Doing presentation in class is quite difficult to me.”
	Embarrassment	When a learner recorded some feeling embarrassed moments in or outside of classrooms, i.e., “It would be a little embarrassing for me to take the initiative to answer questions.”
	Fear	When a learner recorded feeling faced with uncertain or unfavorable conditions and being afraid of negative outcomes which stemmed from personality or pressure (Rahmat, 2020), i.e., “I felt frightened when I was asked a question by the teacher.”
	Indifference	When a learner recorded feeling almost nothing toward any tasks or activities in FC, i.e., “For online classes and exercises in *UNIPUS*, the most frequent emotion I experienced was indifference.”
	Disappointment	When a learner recorded feeling disappointed with some teaching design, activity or task, i.e., “The effect of the *UNIPUS* is really poor.”
	Frustration	When a learner recorded the feeling of being upset or annoyed, especially because of inability to change or achieve something in English learning (Cambridge Dictionary Online), i.e., “I want to learn English well, but I can’t do it.”
	Guilt	When a learner recorded attention toward his or her behavior, negatively scrutinize every aspect of it, and carefully examine ways to undo it, such as ‘if and only if I had [not] done such-and-such’ (Teimouri, 2018), i.e., “I also felt guilty and ashamed when I was distracted and couldn’t answer the question.”

^1^Some scholars use anxiety and nervousness interchangeably, such as Weitoft and Rosén (2005), while others distinguish between the two terms (Hishikawa et al., 2019). Nervousness is a temporary feeling of insecurity associated with specific worries about a stressful situation. These worries usually disappear after someone is successful enough to manage it. Anxiety, on the other hand, is more debilitating and persistent, reflecting recurrent thoughts, as well as negative expectations of events and an inability to tolerate uncertainty. They may be associated with general or specific fears that do not go away despite positive experiences of successfully overcoming them.

### Coding of learner cognition toward flipped classroom

Belkhir (2020, p. 3) stated that the term “cognition” refers to “the process by which knowledge and understanding are developed in the mind.” The adjectival form “cognitive” means “connected with thinking or conscious mental processes.” Cognitive psychologist Matlin (2005, p. 2) defined “cognition” as a mental activity with various cognitive processes. In her view, cognition includes a wide range of mental processes, such as perception, memory, imagery, language, problem-solving, reasoning, and decision-making. She further described the cognitive approach as a theoretical stance that focuses mostly on people’s knowledge and their mental processes. However, for (L2) development from the SCT perspective, social interaction is not just a facilitative mechanism; cognition itself is social (Lantolf and Thorne, 2007). Speech (e.g., the written narrative in the present study), which is, of course, central in SCT, is regarded as an effective tool for thought and action during interaction with either the self and the others (Lantolf and Thorne, 2006). According to Vygotsky’s theory of cognitive development, cognitive abilities are acquired through social instruction and construction, and therefore, learners need to engage in social interactions for L2 development (Vygotsky, 1978). Therefore, in this study, we hold that how the learner perceives a teaching activity or the design of a task in the FC (which is certainly a social activity) renders the “teacher–task–learner” interaction. If a learner recorded his or her perceptions of FC in the narrative (a form of speech), it was regarded as the learner’s cognition toward FC, since perception reflects the individual’s thinking (mental process) of an activity or an event (a social interaction) as discussed above.

### Coding of learners’ activities in flipped classroom

The coding of learners’ activities is only concerned with those activities that the learners participated in and recorded in the narratives, including in and out of the in-person classes both online and offline.

## Results

### Learners’ emotion characteristics and changes in flipped classroom

This section is composed of three subsections that present the participants’ emotional profile at the beginning of and during FC teaching as well as the interaction between emotion, cognition, and activity with the participants’ self-narrative excerpts. (The excerpts are italicized, and pseudonyms are used for the participants).

### Emotional profile at the beginning of flipped classroom teaching

**FIGURE 1:**
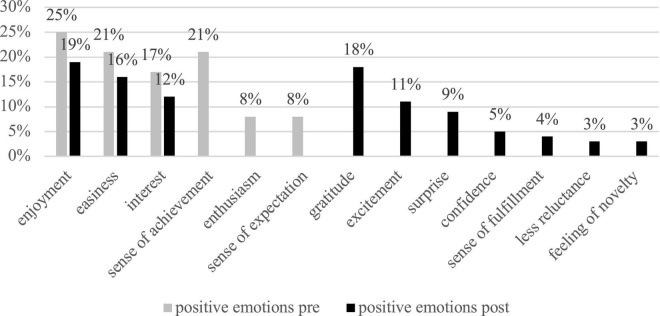
Overall positive emotions from pre- and post-written narratives by the 32 students.

**FIGURE 2:**
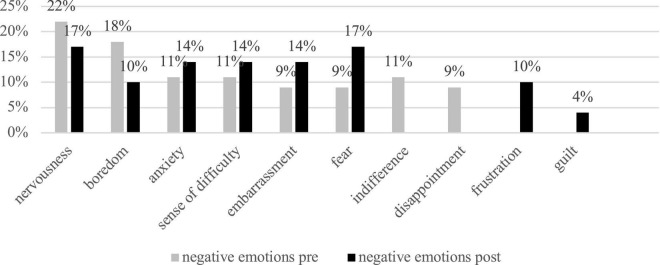
Overall negative emotions from pre- and post-written narratives by the 32 students.

The participants reported experiencing 13 emotions in online and offline mixed FC classes at the beginning of the semester, including five positive-valence emotions and eight negative-valence emotions (see Figures 1, 2 for details). Positive emotions accounted for 35% of all the emotions reported and negative emotions 65%. Among the positive emotions, enjoyment was dominant and was mentioned in 25% of the entries; among the negative emotions, nervousness (22% of the entries), followed by boredom (18%) and anxiety (11%).

### Emotional experiences in the process of flipped classroom teaching

The participants reported a larger portion of positive emotions than negative emotions during the process of FC teaching, although the total number of positive and negative emotions remained unchanged, in comparison to the beginning of this teaching mode (see Figures 1, 2). The dominant positive emotions included enjoyment, gratitude, interest, and excitement; negative emotions included fear, nervousness, embarrassment, sense of difficulty, and boredom. It is worth noticing that boredom decreased from 18% to 10% after FC pedagogy was adopted. Nervousness and anxiety occurred less frequently (see Figure 2). Many new positive emotions like gratitude, excitement, surprise, self-confidence, and sense of fulfillment appeared by the end of the research semester, proving the facilitative function of the FC in positivizing learners’ emotions.

### The unity of learners’ emotion, cognition, and activity

The participants’ self-narratives highlight the unity of learners’ emotion, cognition, and activity, which features both linearity and non-linearity. One way of their combination is that negative emotions led to negative cognition to further negative behaviors. As the case of Vicky, her lower confidence in listening and speaking abilities (cognition) led to her denial of the usefulness of classroom presentation (emotion). Eventually she refused to do the presentation part in the classroom (activity)^1^ :

*As my personal speaking and listening are not very good (C), I may not like the presentation part very much (E), and I don’t know if this thing improves learning (C). I only participated in power point file editing (A). (Excerpt from Vicky’s narrative in week 1*).

Another student, Linda, felt difficulty in learning English well, so she did not take the online course seriously and did not believe in the value of the online lesson. Thus, negative emotion (*I would not take online classes very seriously*) triggered both negative cognition (*I don’t think online lessons can help much for me*) and activity (*Instead of doing homework on the Internet, I prefer paper*) at the same time. In what follows, both negative cognition and negative emotion caused negative behavior. For example, Linda did not understand the words taught by her teacher and felt troublesome to find out their meaning in the dictionary, so she did not pay attention to the online class:

*I am very busy with my major studies (C). I want to learn English very well, but I feel so difficult to do it well (C). I would not take online classes very seriously (E). And, I don’t think online lessons can help much for me (C). Instead of doing homework on the Internet, I prefer paper (A). It is very convenient to mark it (C). Sometimes when I don’t understand the words taught by online teachers and it is troublesome to look it up in the dictionary (E), I would not watch online classes carefully and seriously (E). In general, I really don’t like online classes (E). (Excerpt from Linda’s narrative in week 15).*

Jenny’s data showed that negative emotions could bring positive actions (or activities participated in) and cognition. She felt anxious because of a heavy workload with her major and did not feel satisfactory with her course grades. Both anxiety (emotion) and dissatisfaction (cognition) led to her increased self-planning behaviors (activity) and positive self-concept of planning ability (cognition):

*Due to the heavy work from my major language courses, there may be delays in time plan (C). Before I took English class I concentrated on a lot of things, so I got anxious sometimes (E). After studying English this semester, I feel that I have improved a lot in terms of autonomous learning (C). When I was preparing for the CET 4 exam last semester, I didn’t really study down to earth because I was too ambitious to complete too many tasks at the same time (E). The final grade was not very satisfactory (C). This semester I was preparing for the CET 6 exam, I have set up a daily plan according to my own situation, and memorized words carefully every day (A). Although I don’t know whether my final grades will satisfy me, my self-planning ability has been improved (C). (Excerpt from Jenny’s narrative in week 15).*

There was evidence showing that positive actions contribute to positive emotions and positive cognition. The student, Ryan, answered her teacher’s question well (activity) and was praised by the teacher, leading to her happiness (emotion) and greater engagement (activity) in the English class. Eventually, she believed that even if she would pass the important English test of CET 6 for college students in China and was determined to make more efforts to learn the language (cognition).

*In a Listening and Speaking class, I answered a question very well (A). The teacher said ‘very good’, which made me very excited and happy for the whole class (E). When I felt the homework is boring (E), the teacher still examined our homework with passion and gave us feedback on WeChat group on time (A). I was often ashamed of being called to answer questions (E) because I didn’t preview the contents of the class in time before class (A), but the teacher kept waiting for my reply, reminded me and encourage me (A). So I am willing to learning English and take classes seriously (E). Even if I pass the CET 6, English learning is not over (C).* (*Excerpt from Ryan’s narrative in week 15).*

There was a non-linear flow from positive cognition to positive cognition through positive action and positive emotion. In the case of Ryan, she recognized the teacher’s efforts in preparing the course (activity) and thus took each lesson carefully and was willing to complete homework (emotion). In the end, she thought doing homework was a great training for listening and writing (cognition).

*I understand teacher took a lot of effort in preparing lessons (A), so I take each class very attentively (E). The teacher made me discover the joy of learning English (E). I am willing to complete my homework carefully after class (E). The seemingly boring transcribing homework (E) is actually a great training for listening and writing (C). (Excerpt from Ryan’s narrative in week 15).*

### Dramas accounted for the interaction between cognition, emotion, and activities in flipped classroom teaching

As discussed before, drama is the unit of analysis of a learner’s *perezhivanie*, which refers to “dramatic events” that cause conflicts or change of the learner’s *perezhivanie—*unity of cognition, emotion, and activities. Digging into the data, we found some typical dramas, mentioned later, some of which are common among students, and others are of individual characteristics.

### Dramas of classroom activities

Among a variety of interactive activities, such as “textual structure analysis,” “sentence meaning induction,” and “passage theme extraction,” the participants particularly welcomed those which offered them a chance to be exposed to novel out-of-textbook knowledge, for example, watching movies and book recommendation. All the students perceive these activities helpful and beneficial to English learning (cognition, dramas 1 and 2). The underlined lines in the excerpt of drama 1, “*learning English by watching an excerpt from the classic movie The Devil Wears Prada*” attracted more attention from the learners and added more fun, and their *perezhivanija* or the interaction between activities, emotion, and cognition surely improved their learning motivation. In addition, drama 2 is the classroom activity of “recommending an interesting book.” Jim was extremely interested and serious in this activity. His *perezhivanie* even extended to future improvement of the presentation skills in other courses. Certainly, drama 2 is a very crucial social event in Jim’s learning experience.

Drama 1^2^ :

*In face-to-face classes, there are many interactions between the teacher and students (A), which can mobilize my attention (C). One of the most impressive scenes in this semester is learning English by watching an excerpt from the classic movie ‘The Devil Wears Prada’ (A-Drama). I feel that it adds some fun (E) to combine the boring content of the lesson with the movie clips. (Excerpt from Jenny’ narrative in week 15, June 29, 2021).*

Drama 2:

*When it came to the lesson about reading, the teacher asked the whole class to bring a book they like, write down the reasons to recommend it in English (A-Drama), and then the whole class randomly exchanged their books (A), which is very interesting (E). The teacher also participated and got my book. I was so happy (E). So, when preparing for the presentation, I was quite serious about it (A). It was a relatively smooth presentation for the first time, and I didn’t get stuck (A). Although there will be no English classes in the future, and there will be no English presentations, there will still be presentations in other courses, so it is better to prepare well (C). (Excerpt from Jim’s narrative in week 15, June 29, 2021).*

### Dramas of the teacher contribution

The teacher plays a crucial role in determining learners’ emotional experiences. By selecting well-designed tasks and interesting learning materials, teachers can highly promote their students’ positive emotions. In addition, their emotional support to students, including positive feedback following students’ performance, recognition, and encouragement, provides students a safe psychological environment wherein positive emotions are nurtured. In the case of Ryan, for example, the “dramatic events” related to her *perezhivanie* development is attributed to the responsible teacher who made her discover the joy of learning in the FC because the teacher would prepare “*unique and carefully selected”* materials and videos that she would use in class, which made her learn English more attentively out of interest (see drama 3). Rita had a similar experience of having a good responsible teacher (in previous learning history in the middle school) who led to the change of her *perezhivanie*, which means her learning activities, emotion, and cognition all changed toward positivity because of the teacher (see drama 4).

Drama 3:

*Ryan: “Our teacher is cheerful and full of positive energy, sometimes quite humorous. Her classes have very rich content, from which I have always learned a lot of knowledge (C). The materials and videos prepared in each Listening and Speaking class are unique and carefully selected (A-Drama), not just those materials in the textbook. I understand teacher took a lot of effort in preparing lessons, so I take each class very attentively (C)*…*Whenever I felt the homework is boring (E), I found the teacher still examined our homework with passion (E) and gave us feedback on WeChat group on time (A). “(Excerpt from Ryan’s narrative in week 15, June 28, 2021).*

Drama 4:

*Rita: My English was only 60/100 at a very low level when I was in the primary school, but in the middle school I met a terrific teacher who was very helpful and cared about me very much (A-Drama)*…*I liked him (E) and I think I want to learn English well (C), so I started to like English (A), even now I still enjoy learning English very much (E). (Excerpt from Rita’s 2nd verbal narrative in week 15, July 1, 2021).*

### Dramas of peer contribution

Active peer dynamics in the classroom was conducive to the participants’ positive emotions. They reported that getting new knowledge shared by peers during the time of oral presentation (drama) led to their surprise and enjoyment, which is another drama. By listening to others’ presentation allowed the participants to know their classmates better and thus build inter-personal cohesion, which, in turn, contributed to their positive emotions of a good surprising gain in the class (as shown in drama 5).

Drama 5:

*Jenny: When discussing everyone’s fashion choices (A), I found that everyone has different ideas (C-Drama). I was surprised (E) because I was exposed to different concepts, and I felt that it was also an opportunity to get to know others (C). (Excerpt from Jenny’s narrative in week 15, June 29, 2021).*

### Dramas of previous English learning history

Individual difference plays an important role here (Wang and Derakhshan, 2021). Learners’ levels of language proficiency determine to what extent they take part in classroom activities. Some of the current participants felt interested in their peer classmates’ presentations because their language capability allowed them to understand what was said. On the contrary, those low language proficiency tended to have negative feelings like boredom, apathy, and even helplessness because they neither could personally get involved in class activities nor understood others. Jack’s past English learning history shows he was learning science in high school and his self-awareness of his low proficiency made him lack self-confidence, which led to his disbelief in this class learning helpfulness.

Drama 6:

*Jack: In high school, I studied science (A). Chinese and English have always been drags for me (E-Drama), but after college entrance examination, I came to this university to learn language by mistake. At the beginning, I tried (A), but then I didn’t understand it at all (C). I have been learning Spanish (A), so I have almost forgotten English (C). Now I’m a little bit self-defeating (E). (Excerpt from Jack’s narrative in week 15, June 29, 2021).*

However, Zoey, on the opposite, was a bit unlucky. Once teased by peers on her “strange” English accent turned out to be her drama, which made her puzzled and feared speaking up again in class. To make things even worse, she was confused about the value of classroom learning because some boys in the class would love to make fun of her accent.

Drama 7:

*Zoey: There was a semester in Grade One, every time when I was asked to speak up in the classroom (A), my peers would laugh at my accent (A-Drama)*…*I don’t know why (C), maybe because there were too many boy classmates in the class and they loved to make fun of my accent (A)*…*So ever since then I feared speaking up in class (E & A)*…*(Excerpt from Zoey’s 2nd verbal narrative in week 15, July 1, 2021).*

In a word, learners experienced an array of dramas in the FC, which could be emotional, cognitive, or behavioral “dramatic events” that aroused the interaction of the learner’s emotion, cognition, and activities or (actions taken), hence causing the dynamics of *perezhivanie*.

## Discussion

### Interplay of learners’ emotion, cognition, and actions (or activities)

This study intended to investigate learners’ emotional experiences in the FC and explore the different ways in which emotion, cognition, and action co-exist in this pedagogical context. Regarding the first question, the results showed that the participants experienced both positive and negative emotions in the FC, mirroring the findings in Li et al.’s (2020), Sampson (2020), and Kruk and Pawlak (2022). In comparison to negative emotions, more positive emotions were reported by the participants, particularly enjoyment, interest, confidence, surprise, and gratitude, showing that the FC can lead to emotional positivity, that is, a high ratio of positive to negative emotions (Jeong et al., 2016; González-Gómez et al., 2017). MacIntyre and Vincze (2017) revealed that positivity led to stronger language learning motivation. Thus, the teacher’s goal is never to erase learners’ negative emotions, which is also impossible, as negative emotions are inevitable part of learning. Instead, they should find ways to limit learners’ negative emotions to the point that negative emotions do not overwhelm the positive ones (Jin et al., 2021). To this end, exercising the FC pedagogy seems to be effective.

Narrative analysis also showed that emotion, cognition, and activity did not stand alone but united in a dramatic event (Greenberg and Safran, 1987; Vygotsky, 1994; Lantolf and Swain, 2019; Agnoletto et al., 2021). This finding reflects the construct of *perezhivanie* and suggests that sociocultural theory is a useful theoretical framework to research into emotion, cognition, or activity (Tasker et al., 2010; Golombek and Doran, 2014; Johnson and Worden, 2014; Golombek, 2015; Veresov, 2017; Johnson, 2018, 2021; Johnson and Golombek, 2018). Regarding the pattern of their combination, it was often shown in the qualitative data that negative (positive) emotion, cognition, and activity were interlocked, but occasionally negative emotions can also lead to positive cognition and actions, showing dialectical relations between the three (Vygotsky, 1994). As the case of Jenny, she felt anxious and dissatisfying with course grade, which made her improve self-planning ability and then forge a positive self-concept about herself. The implication is that negative emotions should not terrify learners and teachers (Li et al., 2020; Li, 2021, 2022). The key point is to find ways to manage negative emotions, which thus do not paralyze thinking and behaviors (Jiang and Dewaele, 2019; Jin et al., 2021), but bring positive outcomes to language learning. On this point, students can make use of their own agency to self-regulate their negative emotions, highlighting the importance of developing learners’ proper cognition of negative emotions and training their self-regulatory strategies of emotions.

### Drama as an effective lens to observe the learners’ *Perezhivanija* displaying both historical and developmental characteristics

For the study of the development of individual higher mental functioning, Vygotsky (1997) believed that *perezhivanie* is historical. Accordingly, in this study, we traced participants’ English learning history in the far past (before college), near past (before the present term), and weekly data. We found that dramas that aroused the change of *perezhivanija* can be relative to learners’ past history, present events, or social relations (Swain, 2013; Ng, 2021). In the narrative, Zoey’s drama of being ridiculed by peers because of her accent is similar to the findings in the study by Jiang and Dewaele (2019) in which they found Chinese students have higher levels of foreign language anxiety, which may be attributable to Chinese educational background. This was also in contrast to Grace’s experience in the study by Swain et al. (2015). Instead of being inspired by the experience of being ridiculed like Grace, Zoey in the present study became afraid of speaking English. Golombek and Doran (2014) also claimed that how individuals interpret the lived experience would influence the way they interpret and react to the current situation.

Drama is also developmental over time. For example, a good teacher in the previous learning history exerted its effect on how students get along with English studies in college (narrative of Rita). In addition, drama also arouses future expected experience with learning, such as Jim who believes that the presentation skills learned in English class can be applicable to other subjects. Drama and *perezhivanie* are two essential concepts for understanding how the general genetic law of development works and how the social becomes the individual (Fleer et al., 2017). To be specific, drama brings rich emotions, forming individual’s critical *perezhivanie*, thus influencing individuals’ trajectory of development.

## Implications

This study holds important implications for both research and teaching. This study shows that emotion does not occur alone but is closely combined with cognition and activity in a certain sociocultural context. Therefore, rather than encouraging isolationism, this study indeed advances a systematic perspective to emotion, cognition, and behavior studies incorporating social and relational factors. In this way, the ever-present cognitive approach to foreign language teaching (Swain, 2013) and the newly emerging emotional turn in language development studies (MacIntyre and Gregersen, 2012; Jin and Zhang, 2019, 2021) finally have a common ground on which they can work. In addition, in this study, although negative emotions were reported by the participants with their FC experiences, positive emotions took hold. This suggests that the FC in general is welcomed by the participants. With its mixed teaching modes that combined in-class teaching and out-of-class learning, the FC indeed can bring a brand new teaching and learning ecology in which learners are given more autonomy to explore after class, and teachers serve as a guide to solve the problems encountered by students during their explorations. Nevertheless, we must also note that the negative emotions that the participants reported should not be ignored since they might signal the disadvantages of the FC, which should be addressed by teachers in specific instructional contexts. Gregersen and MacIntyre (2014), inspired by the positive psychology movement, explained that negative emotions are not always bad as they can help learners to eliminate an obstacle, but they can be paralyzing if not properly dealt with during the teaching and learning process (Dewaele, 2015). Finally, what further emerged from the data was that learners prefer some activities specifically designed for the FC, which required them to prepare before class and present in class with all their research. Most of the participants positively commented on the activities they emotionally enjoyed during the in-class time, and this brings more implications to EFL teachers who are working in the FC mode. It is for sure worthwhile to organize more workshops working out more innovative designs compatible with the FC teaching design as it is of great difference to previous traditional classroom teaching (Gao et al., 2022).

## Conclusion

This study established that the FC could effectively improve learners’ positive emotions in foreign language learning, although they might also experience negative emotions under this teaching mode. With no exception, all teaching approaches and methods have their own pros and cons and thus should be tailored to particular groups of students when applied. In addition, this study suggests that an SCT approach to language psychology gives deeper insights into emotions by providing a very useful analytic tool, *perezhivanie*, that sheds light on the complex interactions between emotion, cognition, and activity. Future studies might continue with the current study design but consider exploring in-depth what accounts for the complex interactions between emotion, cognition, and action in both learners and teachers, and in what ways learners and teachers can make these interactions beneficial.

## Limitations and suggestions

This study has limitations. First, this study was only conducted on one class from a university. The number of participants were limited, and there was a lack of heterogeneity among the samples in many aspects like age, gender, and English learning experiences. Therefore, although the findings are quite inspiring, researchers are encouraged to interpret the findings with caution. Second, this study relied on purely the participants’ self-reports regarding their emotional experiences in the FC, based on which the efficacy of the FC was investigated, thus lacking of field observation to triangulate the narrative findings. In addition, the teacher was not included in this study. Thus, the pros and cons of FC teaching could not be viewed from the teacher’s perspective. Last but not least, we developed robust coding systems for meaning units emerged from the narratives on various kinds of emotions, cognition, and activities; the coding, though of referential value, still needs more shaping for future studies.

## Data availability statement

The original contributions presented in this study are included in the article/supplementary material, further inquiries can be directed to the corresponding author/s.

## Ethics statement

The studies involving human participants were reviewed and approved by the Dalian University of Foreign Languages. The patients/participants provided their written informed consent to participate in this study.

## Author contributions

LQ contributed to the draft writing and data organization. LY collected the data and organized the data. YJ wrote the draft. All authors contributed to the article and approved the submitted version.

## Footnotes

1 In this session, the sentences showing activity, emotion or cognition are marked with: (A) represents lines of “activities,” (C) represents “cognition,“(E) represents emotion.

2 In this session, the sentences showing dramas are annotated with underlines; (A) represents lines of “activities,” (C) represents “cognition,” (E) represents “emotion.”

## Appendix

1. ***Direction for pre-semester narrative for all the participants:***

Please describe your previous experience of English learning before college and what you have experienced in college in the flipped classroom. You may talk about events, persons, or any special artifacts that you can remember. Please write minimally 200 words.

2. ***Direction for post-semester narrative for all the participants:***

Please describe your experience of English learning in the flipped classroom during this semester. You may talk about events, persons, or any special artifacts that you can remember. Please write minimally 200 words.

3. ***Direction for weekly narrative for 8 focal students***

Please describe your experience of English learning in the flipped classroom during this week. You may talk about events, persons, or any special artifacts that you can remember. Please write minimally 200 words.
